# Dispensary Teaching in Edinburgh

**Published:** 1906-11-03

**Authors:** 


### Dispensary Teaching in Edinburgh.

### Dispensaries Supported by Voluntary Contributions.

A special feature in the teaching of the Scottish medical schools is the compulsory attendance at one of the large public dispensaries, which provide medical advice for the sick poor and play a very important part in the practical training of the student. This system of public dispensaries reaches its highest development in Edinburgh, where there are five such institutions scattered throughout the poorer quarters of the city, as well as many others dealing with special diseases, with which for the present we are not concerned. These dispensaries are supported by voluntary contributions, and each is managed by its own committee, consisting chiefly of prominent laymen, though usually including a representative of the medical staff. Each dispensary provides free advice at the institution not only for general affections, but also in a variety of special departments, comprising diseases of women and children, of the eye, ear, and throat, while provision is also made for minor surgery and vaccination.

### Outdoor Visitation.

Patients unable to attend the dispensary are also visited at home, and an outdoor maternity department forms an important part of the machinery, while the necessary prescriptions are dispensed either free or at a nominal charge. The medical appointments are all honorary, but the staff receive the students' fees. All the clinics are open to students in their fourth and fifth years of study, but those on special subjects are usually private classes of the medical men in charge, and of those gynæcology, obstetrics, and vaccination are well attended. The general practice of the dispensary is carried on by six medical officers, either physicians or general practitioners, each of whom attends twice weekly so as to see the new patients in turn on one day of the week, and they also superintend the visiting at the patients' homes. The daily clinic within the institution resembles the routine of hospital out-patients' practice, each patient being examined by the physician, assisted by his students, to whom he demonstrates points of interest and instructs them in diagnosis and treatment, including the writing of prescriptions. The patient receives a prescription which is made up while he waits, and is instructed to report himself on his physician's next visiting day or referred to one of the specialists, or, should his condition require it, recommended to the hospital.

### Method of Working.

The addresses of the patients who desire to be visited are then distributed among the students, who, working singly or in pairs, call on them and continue to treat them as long as is necessary. The student is required to at once inform the medical officer to whom he is attached, should the presence of grave symptoms or notifiable infectious disease render it necessary; or should he for any reason desire assistance, his chief is always ready to talk things over or see the patient when required. Minor operations are usually performed by the student under his chief's supervision, while should it be necessary to send the patient to hospital the student arranges for his admission to the wards to which he is attached, and is thus able to follow the subsequent progress of the case. The principle acted on throughout is that of allowing as much freedom to the student as is consistent with the adequate treatment of the patient.

### Sense of Responsibility.

The sense of responsibility acts as an excellent check on rashness of treatment, as for the first time in his career the student is brought into contact with patients under conditions similar to those of private practice. The more acute cases being often sent to hospital owing to the difficulty of otherwise securing proper nursing, many of the remainder suffer from comparatively less serious affections, so that to a student who has carefully followed his lectures and clinical instruction, the difficulties met with are due to inexperience rather than want of knowledge, and rapidly disappear with increasing confidence. All prescriptions pass through the hands of a qualified dispenser before being compounded, so that errors in dosage or compatibility are not only checked, but promptly brought to the notice of the prescriber. The absence of many of the conveniences of hospital practice serves admirably to develop the ingenuity of the students, many of whom become adepts in the art of extemporising appliances for the comfort of their patients.

### The Maternity Department.

The students of the maternity department receive direct supervision in the conduct of their earlier cases, and are instructed to seek assistance in the event of any difficulty or complication. A very large proportion of the midwifery cases available for teaching purposes in Edinburgh are attended through the dispensaries, and therefore the recent report on midwifery teaching presented to the General Medical Council has created some apprehension, as were the suggested regulations carried out great injustice would be inflicted on a number of competent teachers, while the supply of clinical material in this subject would be quite inadequate for the needs of the school.

### Supplements the Students' Work.

It is evident that the dispensary clinic usefully supplements the students' work in the out-patient department of the hospital, in so far that the smaller numbers, both of patients and students, greatly facilitate individual instruction, but it is in the outdoor practice that the importance of dispensary teaching is most apparent. Though "good cases" in the hospital sense may not be of daily occurrence, the student learns much that is of value to him in his subsequent practice. To grasp the main facts of a case with method and tact, to meet difficulties, many of them only indirectly connected with treatment, to answer the queries of anxious friends, and to become familiar with the domestic treatment of minor ailments, are no mean accomplishments, and no amount of hospital training can entirely replace dispensary practice as a field for their acquisition. The direct charge of a number of patients (under proper supervision) is the most satisfactory method of attaining that confidence which is so essential to the successful practice of medicine.

